# Supplementary figures and images for: Autoantigen profiling reveals a shared post-COVID signature in fully recovered and Long COVID patients
Source: medRxiv. 2023 Feb 9:2023.02.06.23285532. Preprint. [Version 2] doi: 10.1101/2023.02.06.23285532 (PMC9934805; doi:10.1101/2023.02.06.23285532)

A

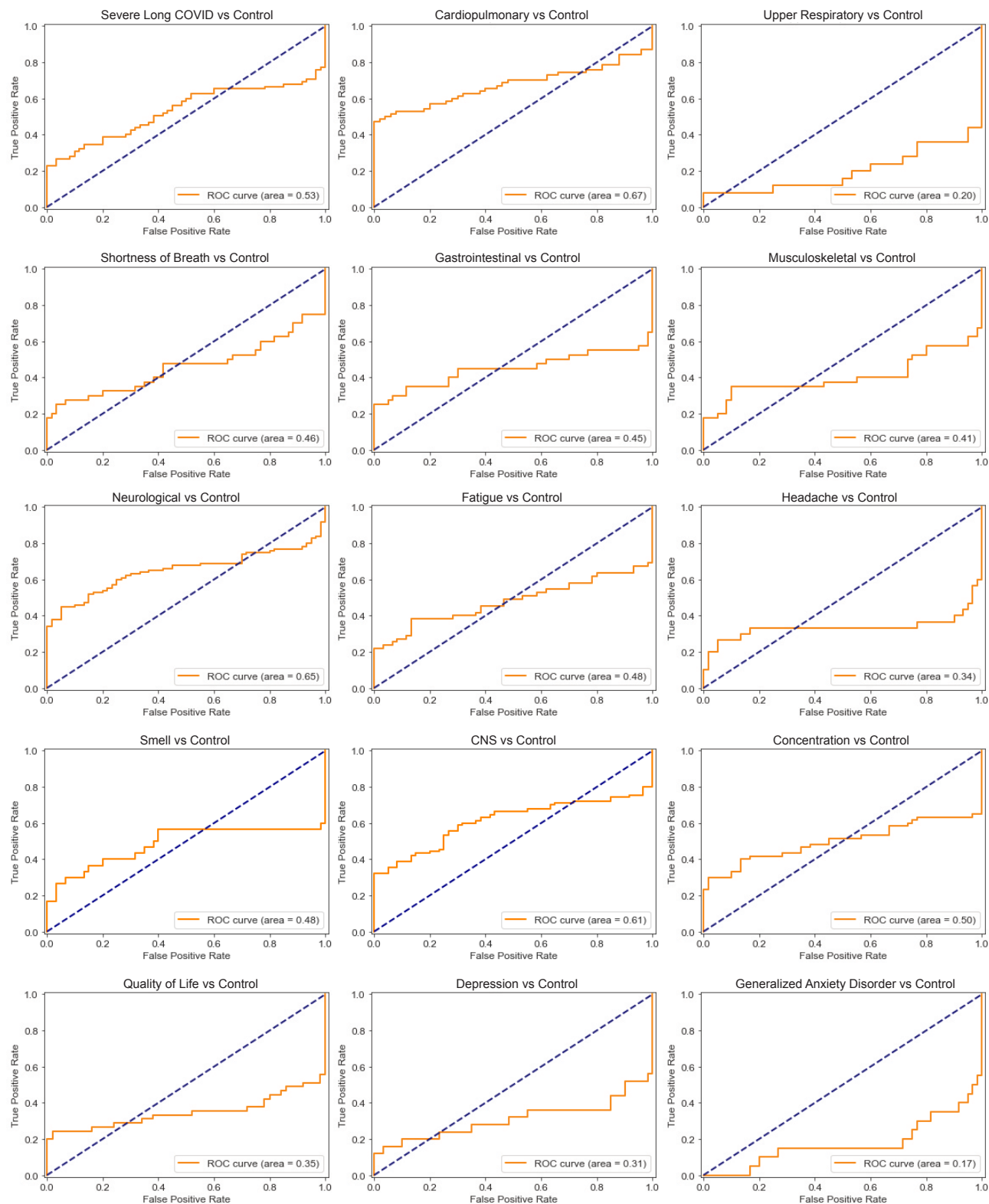

B

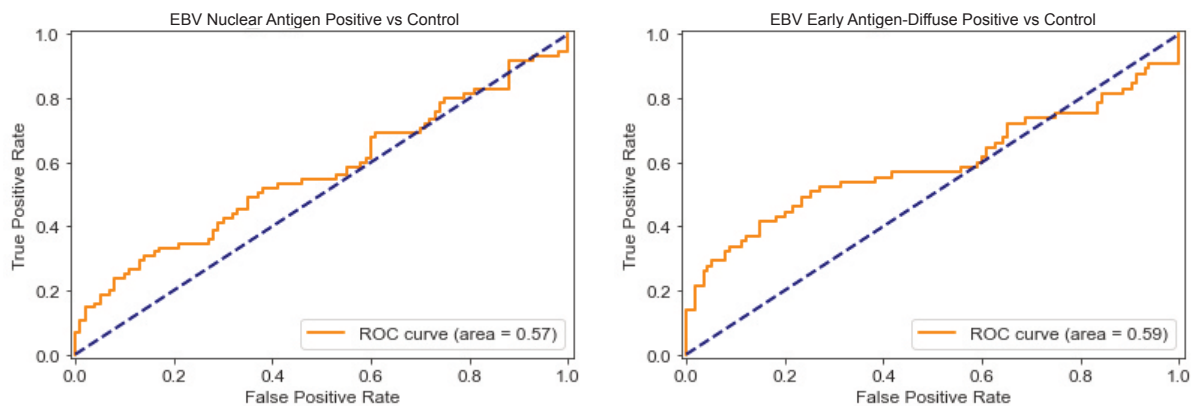

Supplement: Supplement 2 — Supplemental Figure 1: PhIP-Seq is unable to distinguish Long COVID symptom phenotypes from controls. (A) Logistic regression receiver operating characteristic (ROC) curves for Long COVID patients with different specified symptom phenotypes relative to patients previously infected with COVID without the phenotype, or (B) with and without EBV laboratory findings. [file media-2.pdf]
